# Supplementary material for: An enhanced intracellular delivery platform based on a distant diphtheria toxin homolog that evades pre-existing antitoxin antibodies
Source: EMBO Mol Med. 2024 Aug 19;16(10):2638–51. doi: 10.1038/s44321-024-00116-z (PMC11473700; doi:10.1038/s44321-024-00116-z)
Supplement: Supplementary file 2 — Appendix [file 44321_2024_116_MOESM2_ESM.pdf]

APPENDIX

An enhanced intracellular delivery platform based on a distant diphtheria toxin homolog that evades pre-existing anti-toxin antibodies

Shivneet K. Gill[1,2], Seiji N. Sugiman-Marangos[2], Greg L. Beilhartz[2], Elizabeth Mei[3,4], Mikko Taipale[3,4], Roman A. Melnyk*[,1,2]

[1]Department of Biochemistry, University of Toronto, Toronto, ON, Canada, M5S1A8; [2]Molecular Medicine Program, The Hospital for Sick Children Research Institute, 686 Bay Street Toronto, ON, Canada, M5G 0A4; [3]Department of Molecular Genetics, University of Toronto, Toronto ON, M5S1A8, [4]Donnelly Centre for Cellular and Biomolecular Research, University of Toronto, Toronto, ON M5S 3E1, Canada.

Table of Content:

Appendix Table S1: Data collection and refinement statistics (molecular replacement) ……………………………………………………………………3

Appendix Figure S1: Phylogenetic tree of DT homologs………………..……4

Appendix Figure S2: AlphaFold 2.0 error scores…………………………..….5

Appendix Figure S3: Toxicity data of ACT1 and ACT2……………..…………6

Appendix Figure S4: Sequence alignment of key functional residues in receptor binding domain. ………………………………………………………………..….7

Appendix Figure S5: CRISPR/Cas9 screen of DT……………..……………..8

Appendix Figure S6: Toxicity data for DT on SORT1 knockout and overexpressing cells……………………………………………………………………………..9

Appendix Figure S7: Epitope residue conservation between DT and ACT1 or DT and ACT2. …………………………………………………………………………10

Appendix Figure S8: Sequence alignment of key functional residues in translocation domain. ……………………………………………………..……11

Appendix Table S1 Data collection and refinement statistics (molecular replacement)

	ACT1	TpeL-GTD
**Data collection**		
Space group	$P2_12_12_1$	$P12_11$
Cell dimensions		
a, *b*, *c* (Å)	48.24, 138.84, 152.11	75.09, 64.73, 121.18
α, β, γ (°)	90, 90, 90	90, 102.03, 90
Resolution (Å)	102.54–2.50	118.52-2.22
R_{merge}	0.233 (1.146)	0.08 (0.60)
$I / \sigma I$	7.1 (2.0)	11.2 (2.1)
Completeness (%)	100.0 (100.0)	99.4 (100.0)
Redundancy	10.5 (10.6)	4.6 (4.6)
**Refinement**		
Resolution (Å)	51.27 (2.50)	73.49 (2.22)
No. reflections	36,410 (2,613)	56,042 (2,780)
R_{work} / R_{free}	23.62/26.76	19.29/23.74
No. atoms		
Protein	7,802	8,833
Ligand/ion	7	-
Water	409	363
B-factors		
Protein	35.19	48.21
Ligand/ion	71.04	-
Water	32.97	46.43
R.m.s. deviations		
Bond lengths (Å)	0.003	0.007
Bond angles (°)	0.715	1.008

*Number of xtals for each structure should be noted in footnote. *Values in parentheses are for highest-resolution shell.

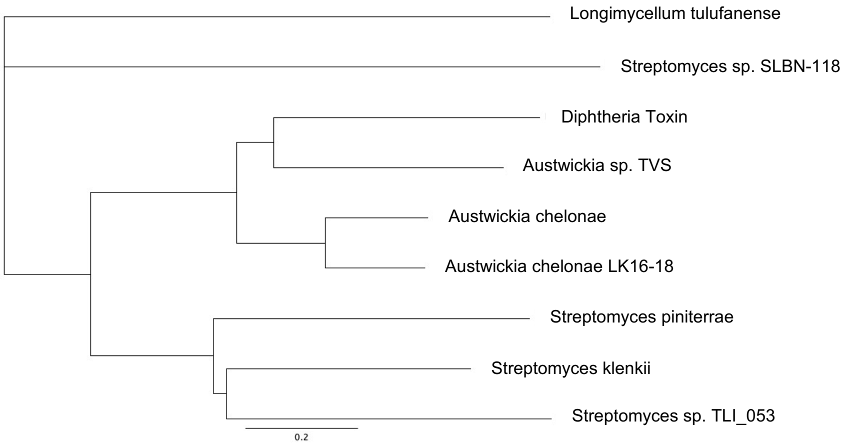

Appendix Fig. S1| Phylogenetic tree of DT homologs. The sequences of each DT homolog were aligned using Blosum62, and a phylogenetic tree was generated using the Geneious 11.0.5 Tree Builder using the Jukes-Cantor genetic distance model and the Neighbour-Joining tree build method. DT homolog proteins are named by the species from which they were extracted. Austwickia genus proteins are the closest to DT.

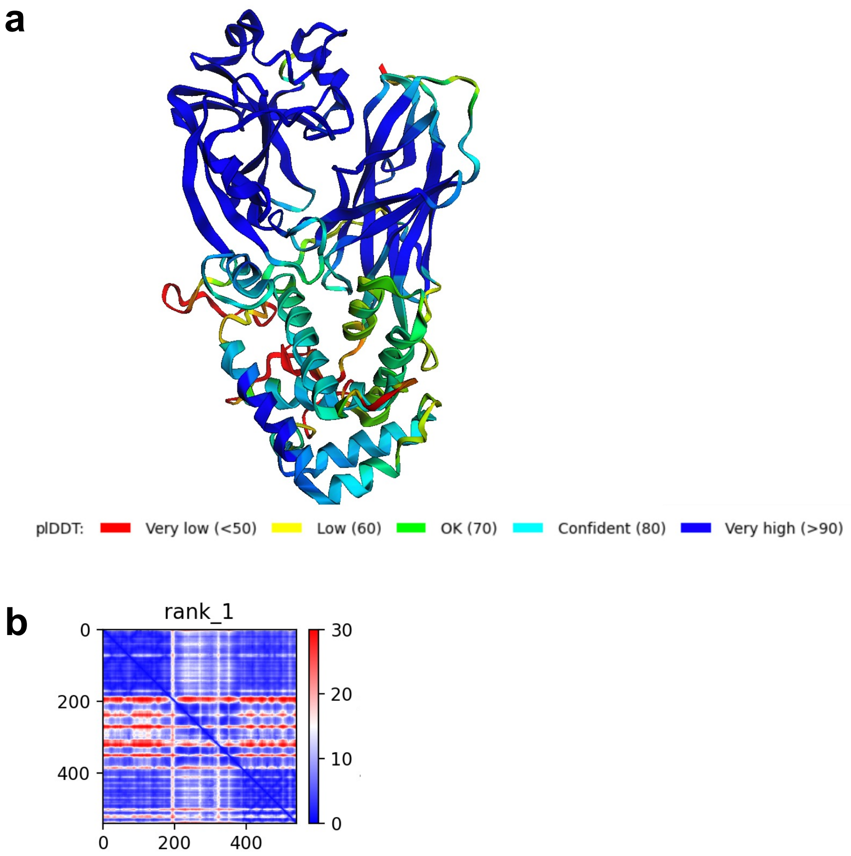

Appendix Fig. S2| AlphaFold 2.0 error scores. The sequence of ACT2 was inputted into the online colabfold notebook. a, Predicted local distance difference test (pIDDT) score for rank 1. The translocation domain is predicted the poorest, and the C- and R-domains are predicted with high probability. The corresponding predicted alignment error (PAE) score for rank1, where blue represents low alignment error, and red represents high alignment error. Overall, there is low error in the structure predicted in rank 1.

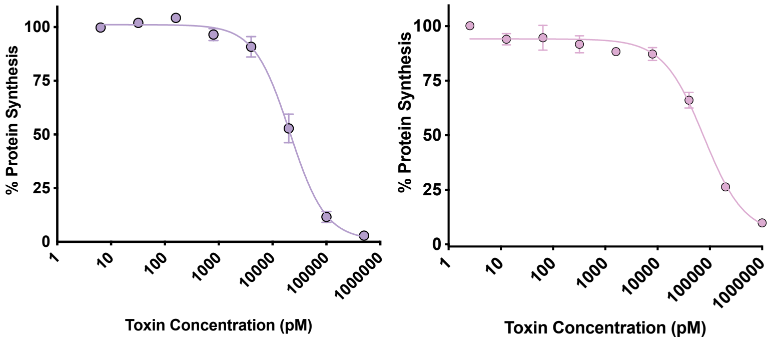

Appendix Fig. S3| Toxicity data of ACT1 and ACT2. Full length ACT1 and ACT2 were purified and tested on Vero-nLucP cells, and luminescence signal was measured at 24 hours post treatment. Both ACT1 (purple) and ACT2 (pink) have EC_{50} values >50nM.

	391	430	433	464	465	468	470	510	512	516	523	526		
	H	A	L	I	D	V	F	G	L	K	V	K	DT	
	S	V	A	T	E	L	F	D	L	I	T	K	ACT1	**% identity** = 23.1%; **% similarity** = 45.0%
	S	T	M	A	G	L	F	D	L	I	T	K	ACT2	**% identity** = 28.0%; **% similarity** = 47.8%

Appendix Fig. S4| Sequence alignment of key functional residues in receptor binding domain. The key residues in DT_R implicated in HB-EGF binding are shown. The corresponding residues in the R domains of ACT1 and ACT2 are shown below. Residues were aligned structurally in pymol. Colour scheme is as follows: blue = hydrophobic; cyan = aromatic; red = positive charge; magenta = negative charge; green = polar; orange = glycine; cyan = aromatic.

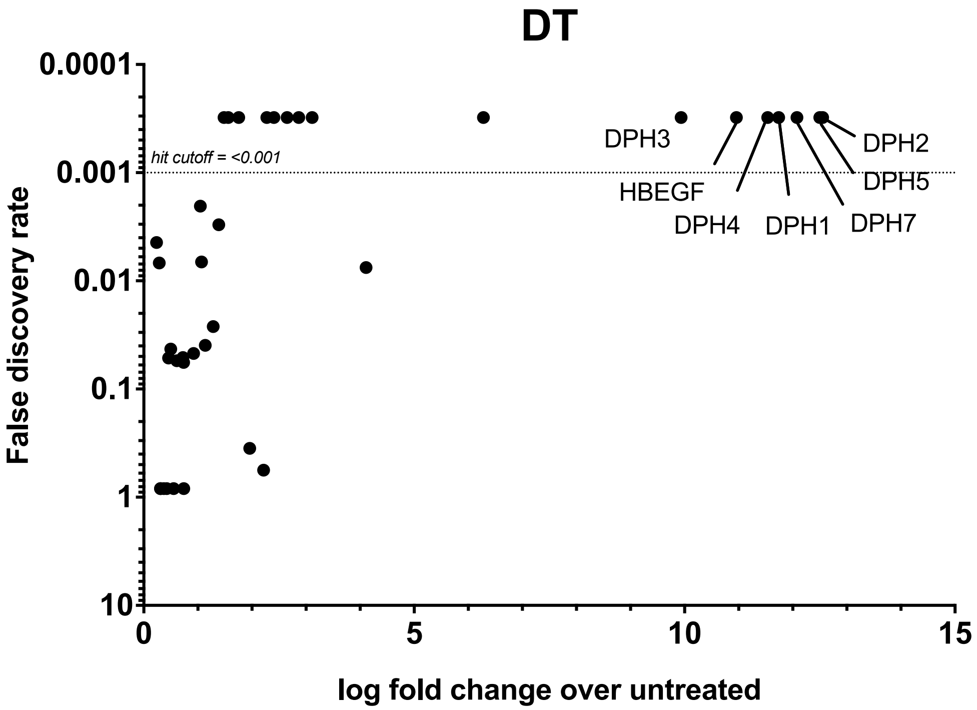

Appendix Fig. S5| CRISPR/Cas9 screen of DT. EC_{99} (10pM) of DT was tested on Hap1 cells, and the top hits were diphthamide synthesis genes, and HBEGF (the receptor).

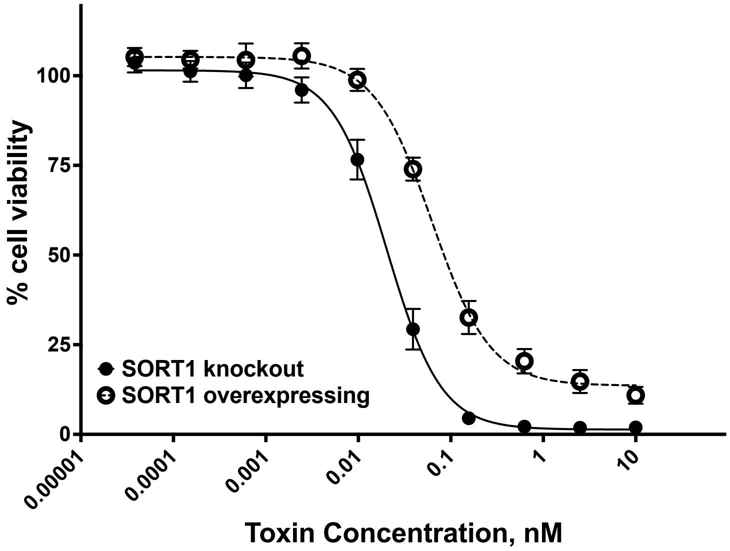

Appendix Fig. S6| Toxicity data for DT on SORT1 knockout and overexpressing cells. DT was titrated on SORT1 knockout and SORT1 overexpressing cells. SEM, n=6.

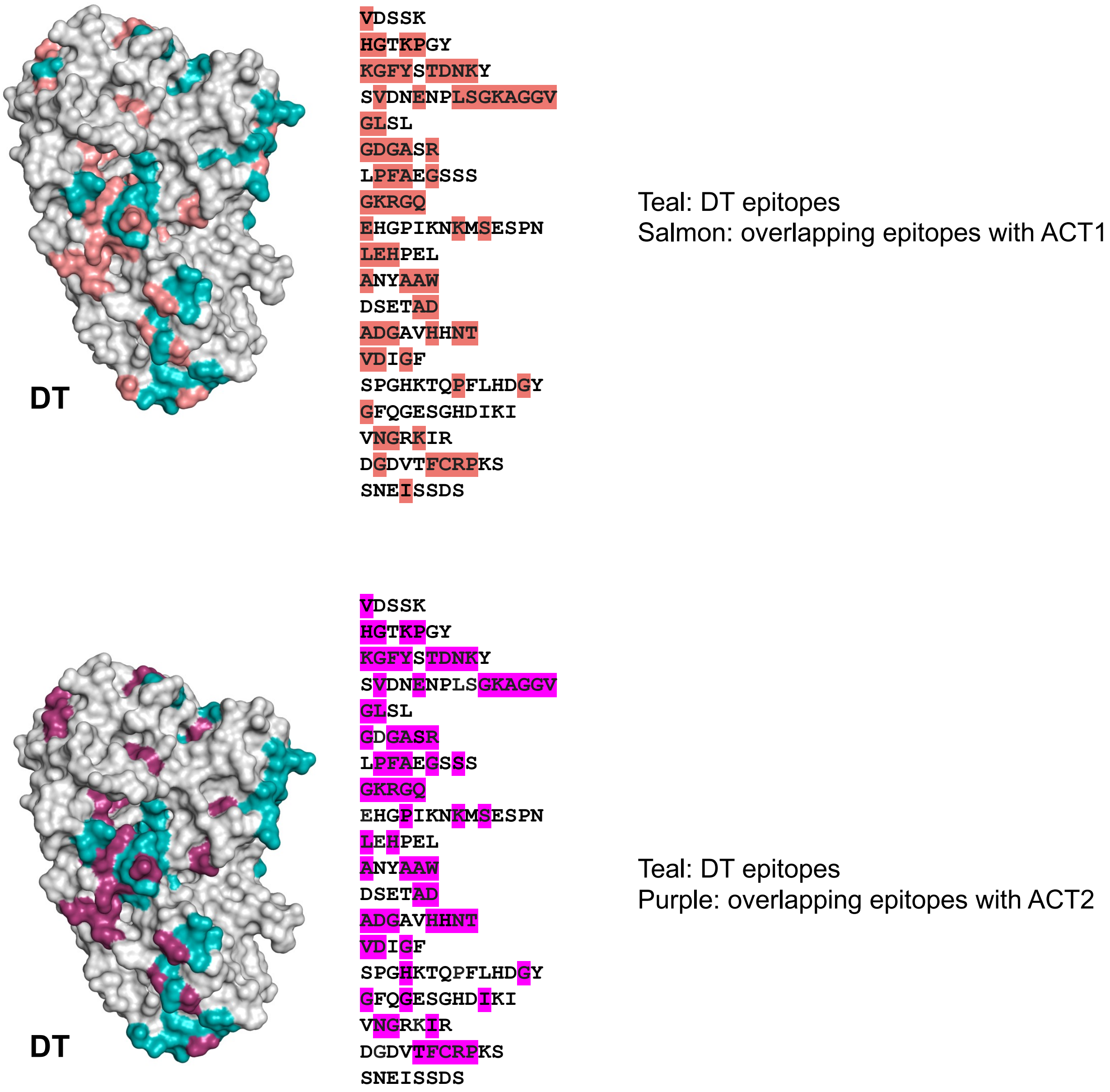

Appendix Fig. S7| Epitope residue conservation between DT and ACT1 (top) or DT and ACT2 (bottom). The residues implicated in B-cell recognition of DT that are not conserved in ACT1 or 2 are highlighted in teal. The residues that overlap with ACT1 are highlighted in salmon (top) and the residues that overlap with ACT2 are highlighted in purple (bottom). ~50% of the epitope residues are conserved, however only one epitope is entirely conserved in both ACT1 and ACT2 (GKRGQ).

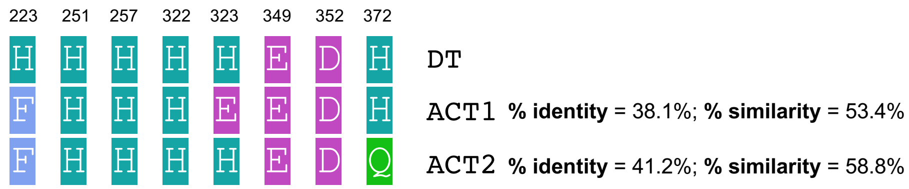

Appendix Fig. S8| Sequence alignment of key functional residues in translocation domain. The key residues in DT_T function are shown. The corresponding residues in the T domains of ACT1 and ACT2 are shown below DT. Residues were structurally aligned in pymol. Colour scheme is as follows: blue = hydrophobic; cyan = aromatic; magenta = negative charge; green = polar; cyan = aromatic.
